# Moral Enhancement Using Non-invasive Brain Stimulation

**DOI:** 10.3389/fnhum.2017.00077

**Published:** 2017-02-22

**Authors:** R. Ryan Darby, Alvaro Pascual-Leone

**Affiliations:** Berenson-Allen Center for Noninvasive Brain Stimulation, Cognitive Neurology Unit at Beth Israel Deaconess Medical Center, Harvard Medical SchoolBoston, MA, USA

**Keywords:** morality, enhancement, brain stimulation, TMS, tDCS, ethics, neuroethics

## Abstract

Biomedical enhancement refers to the use of biomedical interventions to improve capacities beyond normal, rather than to treat deficiencies due to diseases. Enhancement can target physical or cognitive capacities, but also complex human behaviors such as morality. However, the complexity of normal moral behavior makes it unlikely that morality is a single capacity that can be deficient or enhanced. Instead, our central hypothesis will be that moral behavior results from multiple, interacting cognitive-affective networks in the brain. First, we will test this hypothesis by reviewing evidence for modulation of moral behavior using non-invasive brain stimulation. Next, we will discuss how this evidence affects ethical issues related to the use of moral enhancement. We end with the conclusion that while brain stimulation has the potential to alter moral behavior, such alteration is unlikely to improve moral behavior in all situations, and may even lead to less morally desirable behavior in some instances.

## Introduction

Biomedical enhancement refers to biomedical interventions used to improve certain capacities beyond normal, rather than to restore capacities deficient as a result of a disease (Chatterjee, 2004). Cognitive enhancement, for example, involves using biomedical interventions like drugs (Maher, 2008) or non-invasive brain stimulation (Wurzman et al., 2016) to improve an individual's memory, attention, executive functions, or other cognitive functions beyond normal. The use of cognitive enhancement has increased, leading to intense ethical debates about whether such enhancement is morally permissible (Chatterjee, 2004; Greely et al., 2008; Darby, 2010).

Moral enhancement refers to improving moral or social behavior, rather than cognition or physical attributes (Harris and Savulescu, 2014). Morality, broadly defined, refers to evaluating whether an action (or person performing an action) is good or bad (Haidt, 2001). The idea to use biomedical interventions to improve moral behavior is not new. For example, Delgado, an early pioneer in brain stimulation, argued that progress toward a “psycho-civilized society” would require both educational and biomedical interventions to improve moral motivations and reduce tendencies toward violence (Delgado, 1969). His concerns for unrestrained advances in “technologies of destruction” (Delgado, 1969) without accompanying advances in moral behavior parallel many modern ethicists (Persson and Savulescu, 2013). Interventions to treat violent or immoral behavior were also advocated for by early supporters of psychosurgery (Mark, 1970).

Moral enhancement raises a number of unique ethical questions that are different than ethical issues in the use of cognitive enhancement. For example, cognitive enhancement benefits the individual but can be harmful to society in terms of distributive justice and fairness. However, the opposite argument could be made for moral enhancement, which is more likely to benefit society but not necessarily the individual (Douglas, 2016). Second, allowing cognitive enhancement is argued to maximize an individual's autonomy [although coercion to use cognitive enhancement in order to “keep up” may limit autonomy (Darby, 2010)]. In contrast, critics of moral enhancement argue that such interventions would significantly limit an individual's freedom, freedom that is intrinsically valuable even if that choice is to behave immorally (Harris, 2011). Third, the “moral” decision in many situations remains uncertain, and depends on one's philosophical, political, religious, or cultural beliefs. Moral “enhancement” might therefore differ depending on one's ideology. Finally, while proponents of cognitive enhancement argue that cognitive interventions should be morally permissible, certain proponents of moral enhancement argue that moral enhancement should be morally obligatory, or required (Persson and Savulescu, 2013).

A critical point is that discussions about cognitive enhancement often focus on improving cognitive capacities as a means to improve cognition, while discussions in moral enhancement have focused on the ultimate ends of moral enhancement. However, by doing so, the practical means of how altering specific cognitive-affective capacities can lead to changes in moral behavior is neglected. Given the intense and conflicting ethical opinions about moral enhancement, it is especially important to consider whether morality can be enhanced, and, if so, what the likely consequences of such modification will be Douglas (2013). We will argue that morality does not consist of a single neuropsychological capacity, but rather is a group of neuropsychological capacities that guide normal social behavior (Darby et al., 2016). This position is not new, and is based on a growing body of evidence from moral psychology (Cushman et al., 2006; Cushman and Young, 2011), functional neuroimaging (Greene et al., 2001, 2004; Young et al., 2007), and the study of neurological patients with antisocial behaviors (Moll et al., 2005; Ciaramelli et al., 2007, 2012; Koenigs et al., 2007; Mendez, 2009; Thomas et al., 2011; Fumagalli and Priori, 2012; Ibañez and Manes, 2012) demonstrating the complexity and heterogeneity of the “moral” brain.

In this article, we review the existing literature on the use of non-invasive brain stimulation (NIBS) to modulate moral behavior. Rather than improving or impairing a single “moral” process, we will show that non-invasive brain stimulation alters specific neuropsychological processes contributing to normal moral behavior. Such alterations can be viewed as moral “enhancement” in certain situations, but may lead to immoral behavior in other situations. Finally, we will discuss how the evidence from non-invasive brain stimulation affects the ethical debate on moral enhancement.

## Methods

We searched Pubmed (from indexing through 1/2016) for all articles involving non-invasive bran stimulation (using the terms “transcranial magnetic stimulation” OR “transcranial direct current stimulation” OR TMS OR tDCS OR “non-invasive brain stimulation” OR “theta burst stimulation” OR TBS) and moral behavior (using the terms moral OR morality OR altruism OR cooperation OR fairness OR unfairness OR empathy OR social OR aggression). We additionally searched references in review articles for additional cases. This search resulted in 470 articles. From these, 48 specifically involved the use of non-invasive brain stimulation on morally relevant behaviors. We classified articles based on the methodology (neurophysiological response to moral stimuli using NIBS vs. modulation of moral behavior using NIBS), NIBS parameters (TMS vs. tDCS, stimulation, frequency, duration, location, use of neuronavigation), psychological tests used, and significant findings.

### Modulation of moral judgments using non-invasive brain stimulation

A small but growing number of studies have examined the role of non-invasive brain stimulation in moral behavior and decision-making in normal persons (Table 1). Repetitive transcranial magnetic stimulation (rTMS) studies use low frequency rTMS to create a “functional lesion” to test hypotheses regarding whether specific brain regions are necessary for specific moral judgments, while high frequency rTMS is thought depolarize or activate cortex. Similarly, transcranial direct current stimulation (tDCS) studies examine both whether increasing (anodal) or decreasing (cathodal) cortical excitability in a particular brain region altered moral behavior. However, the actual physiological effects of specific brain stimulation parameters remain unknown. Therefore, it is most appropriate to state that a particular NIBS protocol modulates moral behavior while remaining agnostic as to whether this is due to increasing or decreasing brain activity in a specific region vs. a more complex pattern of modulation. The majority of studies focus exclusively on the role of the dorsolateral prefrontal cortex (DLPFC), although a smaller number have investigated the role of the right temporal-parietal junction (TPJ) and the medial frontal lobe. Finally, it should be noted that this review is likely to under-report non-significant findings given publishing biases toward positive results.

**Table 1:** **Modulation of moral behavior using non-invasive brain stimulation**.

**Study**	**Brain stimulation**	**Localization**	**Significant findings**
**MODULATION WITH tDCS**			
Sellaro et al., 2015b	Anodal/Cathodal/Sham	R. TPJ	Anodal stimulation of R. TPJ led to diminished moral blame for accidental harms
	Intensity of 1 mA	Target: CP6, 35 cm^2^	
	Duration of 20 min	Ref. L. SO, 35 cm^2^	
	Testing post-stimulation		
Sellaro et al., 2015a	Anodal/Cathodal/Sham	mPFC	Anodal stimulation of mPFC reduced racial bias on IAT for reaction time and errors (Cohen's *d* = 0.99)
	Intensity of 1 mA	Target: CPz, 35 cm^2^	
	Duration of 20 min	Ref. Oz, 35 cm^2^	
	Testing during last 10 min		
Kuehne et al., 2015	Anodal/Cathodal	L. DLPFC	Anodal stimulation of L. DLPFC led to LESS utilitarian responses to hard personal dilemmas
	Intensity of 1 mA	Target: F3, 35 cm^2^	
	Duration of 20 min	Ref. P4, 35 cm^2^	
	Testing during last 10 min		
Kelley et al., 2015	Anodal/Cathodal/Sham	L. or R. DLPFC	Anodal stimulation of L. DLPFC stimulation increased jealousy ratings relative to right and sham after social exclusion
	Intensity of 2 mA	Target: F3 or F4, 35 cm^2^	
	Duration 15 min	Ref: F3 or F4, 35 cm^2^	
	Testing post-stimulation		
Nihonsugi et al., 2015	Anodal/Sham	R. DLPFC	Anodal stimulation of R. DLPFC increases trust/cooperation
	Intensity 2 mA	Target: MNI (44, 34, 22), 35 cm^2^	
	Unknown Duration		
	Testing after 5 min	Ref: Oz, 35 cm^2^	
Dambacher et al., 2015b	Anodal/Sham	R. DLPFC	Anodal stimulation of R. DLPFC reduced proactive aggression in men only (Cohen's *d* = 1.5)
	Intensity of 2 mA	Target: F4, 35 cm^2^	
	Duration 12.5 min	Ref: L. SO, 35 cm^2^	
	Testing during stimulation		
Wang et al., 2014	Anodal/Cathodal/Sham	L. DLPFC	Anodal stimulation of L. DLPFC increased empathic pain ratings
	Intensity of 2 mA	Target: F3, 35 cm^2^	
	Duration of 5 min	Ref: FP4, 25 cm^2^	
	Testing post-stimulation		
Riva et al., 2015b	Cathodal/Sham	R. VLPFC,	Cathodal stimulation to R. VLPFC increased negative feelings associated with exclusion (Cohen's *d* = 0.8)
	Intensity of 1.5 mA Duration of 20 min	Target: F6, 25 cm^2^	
	Testing during last 15 min	Ref: L. SO, 35 cm^2^	
Riva et al., 2015a	Anodal/Sham	R. VLPFC,	Anodal stimulation of R. VLPFC decreased aggression after social exclusion (Cohen's *d* = 0.62)
	Intensity of 1.5 mA Duration of 20 min	Target: F6, 25 cm^2^	
	Testing during last 15 min	Ref: L. SO, 35 cm^2^	
Riva et al., 2012	Anodal/Sham	R. VLPFC,	Anodal stimulation of R. VLPFC decreased hurt feelings (Cohen's *D* = 1.04) and unpleasantness (Cohen's *d* = 0.91) after social exclusion
	Intensity of 1.5 mA Duration of 20 min	Target: F8, 25 cm^2^	
	Testing during last 15 min	Ref: L. SO, 35 cm^2^	
Ruff et al., 2013	Anodal/Cathodal/Sham Intensity of 1 mA	R. VLPFC	1. Anodal stimulation of R. VLPFC increases (+33.5%), while cathodal decreases (–22%), giving in ultimatum game, 2. Opposite effect in the dictator game, where cathodal stimulation increases giving, while anodal stimulation decreases giving
	Unknown Duration	Target: MNI (52, 28, 14), 35 cm^2^	
	Testing during stimulation	Ref. Cz, 35 cm^2^	
Mameli et al., 2010	Anodal/Sham	Bilateral DLPFC Target: F3/F4, 32 cm^2^ each	Anodal stimulation to bilateral DLPFC decreased reaction times to making lies about general knowledge (Cohen's *d* = 1.5)
	Intensity of 2 mA		
	Duration of 15 min	Ref. R. deltoid, 64 cm^2^	
	Testing post-stimulation		
Fumagalli et al., 2010	Anodal/Cathodal	Bilateral mPFC	Anodal stimulation of the bilateral mPFC increased, and cathodal decreased, utilitarian judgments in females only
	Intensity of 2 mA	Target: “above eyes,” 54 cm^2^	
	Duration 15 min	Ref. R. deltoid, 64 cm^2^	
	Testing post-stimulation		
Karim et al., 2010	Anodal/Cathodal/Sham Intensity of 1 mA	Anterior PFC	Cathodal stimulation of R. DLPFC improved lying score, reduced reaction time, and reduced guilt of lying
	Duration of 18 min	Target: FP2, 24 cm^2^	
	Testing after 3 min	Ref: PO3, 24 cm^2^	
Hortensius et al., 2012	Anodal/Cathodal/Sham Intensity of 2 mA	R. or L. DLPFC	Anodal Stimulation of L. DLPFC/Cathodal stimulation of R. DLPFC increased aggressive responses
	Duration 15 min	Target: F3 or F4, 35 cm^2^	
	Testing post-stimulation	Ref. F3 or F4, 35 cm^2^	
Priori et al., 2008	Anodal/Cathodal/Sham	Bilateral DLPFC	Anodal stimulation of bilateral DLPFC prolonged reaction times to lies (Cohen's *d* = 2.57)
	Intensity of 1.5 mA	Target: F3/F4, 32 cm^2^ each	
	Duration of 10 min	Ref. Deltoid, 64 cm^2^	
	Testing post-stimulation		
Knoch et al., 2008	Cathodal/Sham	R. DLPFC	Cathodal stimulation of R. DLPFC increased acceptance of unfair offers (+21.2%), without changing judgments of fairness, in ultimatum game
	Intensity of 1.5 mA	Target: F4, 35 cm^2^	
	Duration of 10 min	Ref. L. SO, 100 cm^2^	
	Testing after 3 min		
Civai et al., 2015	Cathodal//Sham	Bilateral mPFC	Cathodal stimulation of mPFC increases rejection of unfair offers in ultimatum game when playing for a third party
	Intensity of 2 mA	Target: MNI (–2, 58, 8), 35 cm^2^	
	Duration of 20 min	Ref: R. arm, 35 cm^2^	
	Testing after 2 min		
Fecteau et al., 2013	Anodal/Cathodal/Sham	R. and L. DLPFC	Both stimulation conditions increased lie generation compared with sham stimulation
	Intensity of 2 mA	Target: F3 or F4, 35 cm^2^	
	Duration of 20 min	Ref: F3 or F4, 35 cm^2^	
	Testing post-stimulation		
Boggio et al., 2009	Anodal/Sham	L. DLPFC, M1, V1	L. DLPFC anodal stimulation reduced unpleasantness (−5.8%) and emotional discomfort (−8.9%) to viewing pain in others
	Intensity of 2 mA	Target: F3, C3, or Oz, 35 cm^2^	
	Duration of 5 min	Ref: R. SO, 35 cm^2^	
	Testing during session		
Rêgo et al., 2015	Anodal/Cathodal/Sham	L. or R. DLPFC	L. DLPFC cathodal/R. DLPFC anodal reduced emotional valence and arousal ratings to viewing pain in others
	Intensity of 2 mA	Target: F3 or F4, 35 cm^2^	
	Duration of 15 min	Ref: F3 or F4, 35 cm^2^	
	Testing after 5 min		
Dambacher et al., 2015a	Anodal/Cathodal/Sham	L. or R. DLPFC	No effects of either stimulation paradigm on Taylor aggression paradigm
	Intensity of 1.5 mA	Target: F7 or F8, 35 cm^2^	
	Duration of 22 min	Ref: F7 or F8, 35 cm^2^	
	Testing post-stimulation		
Wang et al., 2016	Anodal/Sham	R. Orbitofrontal	Anodal stimulation to R. orbitofrontal cortex increased trust/giving in trust game (+15%)
	Intensity of 2 mA	Target: FP2, 9 cm^2^	
	Duration of 15 min	Ref: F4, 9 cm^2^	
	Testing post-stimulation		
Ye et al., 2015	Anodal/Cathodal/Sham	R. or L. TPJ	1. Anodal L. TPJ/ cathodal R. TPJ reduced blame for attempted but unsuccessful harms. 2. Anodal R. TPJ/ cathodal L. TPJ increased blame for successful harms
	Intensity of 2 mA	Target: CP5 or CP6, 35 cm^2^	
	Duration of 20 min	Ref: CP5 or CP6, 35 cm^2^	
	Testing after 15 min		
Sowden et al., 2015	Anodal/Active Sham	R. TPJ vs. Occipital	Anodal R. TPJ improved detection of lying in others (6.8%)
	Intensity of 1 mA	Target: CP6 or Oz, 35 cm^2^	
	Duration of 20 min	Ref: Vertex, 35 cm^2^	
	Testing post-stimulation		
**MODULATION WITH TMS**			
Jeurissen et al., 2014	3-pulses, 150 ms apart	R. DLPFC, R. TPJ Target: Talaraich (39, 47, 7) or (60, −40, 19)	1.3-pulse inhibition at 2.5 s of the R. DLPFC DECREASED utilitarian decisions to personal dilemmas. 2. 3-pulse inhibition at 2.5 s of R. TPJ DECREASED utilitarian decisions to impersonal dilemmas
	Intensity of 70% machine output		
	Stimulation 1.5, 2, 2.5, or 3 s into decision		
Balconi and Canavesio, 2014	TMS 10 Hz vs. Sham Intensity of 120% RMT Duration of 1 s per trial for total of 80 trials	L. DLPFC	10 Hz TMS of L. DLPFC increased decision to help in all scenarios except neutral
		Target: Talairach (–1, 45, 15)	
Baumgartner et al., 2014	rTMS 1 Hz vs. Sham Intensity of 110% RMT Duration of 20 min, Testing post-stimulation	L. TPJ or R. TPJ	1 Hz TMS of R. TPJ decreased punishment of outgroup persons
		Target: MNI (−45, −60, 21) or (57, −60, 30)	
Tassy et al., 2012	rTMS 1 Hz vs. Sham Intensity of 54% stimulator output	R. DLPFC	1 Hz TMS of R. DLPFC decreased utilitarian responses to high conflict personal moral dilemmas (OR 0.248)
	Duration of 15 min	Target: Talairach (45, 36, 24)	
	Testing post-stimulation		
Young et al., 2010	1. rTMS 1 Hz vs. control Intensity of 70% machine output	R. TPJ	1.1 Hz TMS of R. TPJ increased permissibility for attempted but unsuccessful harms. 2. Temporary inhibition of R. TPJ increased permissibility for attempted but unsuccessful harms
	Duration of 25 min	Target: MNI (60, −54, 34)	
	Testing post-stimulation	Control stimulation 5 cm posterior to this region	
	2. TMS 10 Hz vs. control Intensity of 60% machine output		
	Duration of 500 ms at beginning of each judgment		
Knoch et al., 2006	rTMS 1 Hz vs. Sham	R. or L. DLPFC, Target: Talaraich (±39, 37, 22)	1 Hz rTMS of R. DLPFC increased acceptance of unfair offers in ultimatum game (+35.4%)
	Intensity of 54% machine output		
	Duration of 15 min		
	Testing post-stimulation		
Buckholtz et al., 2015	rTMS 1 Hz vs. Sham Intensity of 30% machine output	R. or L DLPFC, Target: Talairach (±39, 37, 22)	1 Hz TMS of either R. or L. DLPFC reduced punishment for responsible moral violators, without changing judgments of blameworthiness, or responsibility (Cohen's *d* = 3.93)
	Duration of 30 min		
	Testing post-stimulation		
Perach-Barzilay et al., 2013	cTBS (3-pulse 50 Hz, delivered at rate of 5 Hz) vs sham, intensity 100% aMT, duration 1 min, testing post-stimulation	R or L. DLPFC	R. DLPFC cTBS reduces both reactive and proactive aggression (Cohen's *d* = 1.62)
	Target: 5 cm anterior to motor hot-spot		
Strang et al., 2015	rTMS 1 Hz vs. Sham	R. or L DLPFC, Target: Talairach (±39, 37, 22)	1.1 Hz rTMS to R. DLPFC reduced giving in both dictator and ultimatum. 2. 1 Hz rTMS to R. DLPFC reduced punishment for low offers
	Intensity of 110% RMT		
	Duration of 15 min		
	Testing post-stimulation		

#### Modulation of specific moral behaviors

One important and well-studied factor in moral behavior is the aversion to violating the personal rights of other persons, such as causing them physical harm. One method of experimentally testing aversion to harming others has been to use personal and impersonal moral dilemmas, where a subject must chose whether to harm one person in order to save many others (Greene et al., 2001, 2004). These dilemmas require choosing between competing moral considerations: the personal rights of a single person, and the aversion to causing direct harm to them, versus the utilitarian benefit of saving a larger number of persons. One Hertz rTMS over the right DLPFC increased utilitarian responses to first-person, high conflict dilemmas specifically, suggesting a diminished aversion to violating the rights of others (Tassy et al., 2012). A potentially conflicting result was seen in a study where three repetitive pulses administered to the right DLPFC at critical time-points resulted in decreased utilitarian responses, suggesting either a disruption in utilitarian considerations, or an enhanced consideration of the personal rights of others (Jeurissen et al., 2014). However, the time-specific protocol of this study in relation to the final moral judgment makes it difficult to interpret the causal role the right DLPFC in either of these processes. It may be that the right DLPFC instead integrates both harm aversion and utilitarian consequences, so that inhibition of a critical time-point would prevent the input of the emotional salience of personal harms from reaching the right DLPFC. This highlights the uncertainty of determining the actual neurophysiologic effects of different types of brain stimulation.

Complicating matters further, anodal stimulation to the left DLPFC led to decreased utilitarian responses (or an increased sensitivity to moral harms) (Kuehne et al., 2015), while in a different study, bilateral anodal stimulation of the medial prefrontal cortex increased, while cathodal stimulation decreased, utilitarian responses in females only (Fumagalli et al., 2010). It therefore remains uncertain whether modulation in this task is based on right vs. left hemisphere stimulation, medial vs. lateral stimulation, or a combination of these factors. Moreover, given that the task leads to competing moral considerations, it is unclear which factor (harm aversion or utilitarian reasoning) is being modified by brain stimulation.

Non-invasive brain stimulation can also influence altruism, trust, cooperation, and other prosocial behaviors. One approach to studying prosocial behavior uses economics games where one person (player A) is given a sum of money and has the option to give a portion of that money to another person (player B). In the dictator game, there is no threat of retribution for unfair offers, while in the ultimatum game, player B can reject an unfair offer, in which case neither player will receive any money (in a sense, paying to punish the unfair offer of player A). Anodal tDCS to the right DLPFC led to lower offers without the threat of punishment but higher offers with the threat of punishment, while cathodal tDCS had the opposite effect (Ruff et al., 2013). However, in a different study, 1 Hz rTMS to the same region reduced giving in both the dictator and ultimatum games (Strang et al., 2015). In another economic game, player A can choose to “trust” player B by giving money to player B, which will be tripled. Player B can then give back a portion of this investment to player A (“cooperate”), or keep an even larger sum of money and not give any money back to player B. Anodal stimulation of the right DLPFC [or the right orbitofrontal cortex (Wang et al., 2016)] increased decisions to trust (as player A) and cooperate (as player B), indicating a general enhancement of prosocial behavior (Nihonsugi et al., 2015). Similarly, 10 Hz rTMS to the right DLPFC enhanced the willingness to intervene to help others in simulated situations (Balconi and Canavesio, 2014). Cathodal stimulation of the right DLPFC increased lying, a behavior where one is being untrustworthy (Karim et al., 2010). These results generally support the view that increasing activity in the right DLPFC increases decisions to trust and cooperate, although reduced giving in the dictator game in one study (Ruff et al., 2013) suggests that this may not be due to pure altruism *per se*, but instead may be due to an increased adherence to expected social norms. Understanding the social expectations of other players in these economic games can lead to the mutually beneficial outcomes available through cooperation compared with selfish playing.

A related issue is whether to punish others who are unfair or violate other social norms. Both low frequency rTMS (Knoch et al., 2006) and cathodal tDCS (Knoch et al., 2008) of the right DLPFC increase a subjects willingness to accept unfair offers as player B in the ultimatum game, suggesting that the perceived unfairness of the offer (which remains unchanged by stimulation) exerts less influence on decision-making. In other words, participants are less willing to “pay” to punish those who give unfair offers. In a model of determining blame and punishment for those committing crimes, simulating the experience of jurors in a criminal trial, 1 Hz rTMS to the right DLPFC reduced the punishment, without affecting blameworthiness, of the criminals (Buckholtz et al., 2015). In contrast, reducing right DLPFC activity (by applying anodal tDCS to the left and cathodal to the right DLPFC) increased punishment of others who were critical to subjects (Hortensius et al., 2012). Retributive punishment after being socially excluded in a virtual ball-sharing task is reduced after anodal stimulation to the right DLPFC (Riva et al., 2015a), as is proactive aggression without any provocation (Dambacher et al., 2015b). These latter findings suggest that the right DLPFC may play a more general role in tempering aggressive behaviors toward others.

Finally, several studies have investigated modulation of the right temporal-parietal junction (right TPJ), an area important in mediating the effects of intentions and mental states on moral judgments, a process referred to as theory of mind (Young et al., 2007). Low frequency rTMS or cathodal tDCS to the right TPJ reduces the effect of the agent's negative belief on moral blameworthiness for attempted but unsuccessful harms (e.g., attempted murder) (Young et al., 2010; Ye et al., 2015), while anodal tDCS to this area resulted in either increased blame for actual harms (Ye et al., 2015), or an increased consideration of an agent's innocent intentions in accidental harms (e.g., accidentally shooting someone), reducing judgments of moral blame (Sellaro et al., 2015b). These results are consistent with neuroimaging literature showing that this area is particularly sensitive to the role of intentions in moral judgments (Young et al., 2007; Koster-Hale et al., 2013). The right TPJ is also involved in biasing in-group vs. out-group status, as inhibition of this region reduces the increased punishment that normally occurs when out-group members do not cooperate compared with in-group members (Baumgartner et al., 2014). A review of how brain stimulation to the right TPJ affects social behavior outside the domain of morality can be found elsewhere (Donaldson et al., 2015; Mai et al., 2016).

#### Neuroanatomical specificity of brain stimulation on moral behavior

Based on these studies, tentative proposals can be mad for the regionally specific effects of brain stimulation on moral behavior.

Inhibition of the right DLPFC reduces the influence of harm on decision-making, both when deciding to perform a harmful action oneself, and when punishing harmful or unfair behaviors in others. This reduces decisions to punish or disapprove of potentially harmful personal moral violations in others. However, it also increased the likelihood of performing a harmful action oneself, such as lying or reacting aggressively. In contrast, excitatory brain stimulation to the right DLPFC reduced aggression and increased adherence to social norms. Taken together, these results suggest that the right DLPFC is important for representing aversion to harm, leading individuals to avoid harming others, but also to punish those who do cause harm.

This role may not be specific to the right hemisphere, as stimulation of the left DLPFC may also results in increased cooperation and increased consideration of harm violations in moral dilemmas.

Finally, the right TPJ presumably exerts a more fundamental role in modulating the role of beliefs and intentions in moral judgments. This may influence the degree to which a harmful action is perceived as moral (e.g., intended) vs. non-moral (e.g., an accident). It also more generally shifts focus toward the viewpoint of other persons, leading to improved lie detection, for example Sowden et al. (2015).

### Moral enhancement and non-invasive brain stimulation

The previous section provides evidence to support our hypothesis that non-invasive brain stimulation can modulate certain cognitive-affective capacities involved in moral cognition. In the following section, we will discuss how modulating capacities contributing to moral behavior, rather than assuming enhancement of a single moral capacity, alters key ethical considerations in moral enhancement.

#### Autonomy

Autonomy refers to the capacity of a person to define his or her preferences, desires, values, and ideals (Dworkin, 1988). A key component of such autonomy is the ability to critically reflect upon, and either accept or change, one's beliefs (Dworkin, 1988). Proponents of cognitive enhancement argue that individuals should be allowed to use cognitive enhancement if such use aligns with their values and beliefs (Darby, 2010). A similar argument can be made for moral enhancement. Moral enhancement allows for an individual to express his or her autonomy by choosing to improve specific capacities contributing to moral behavior. For example, brain stimulation could potentially modulate one's capacity for empathy, or one's predisposition toward reacting aggressively. However, as discussed previously, such modulation is likely to affect only certain moral behaviors, but not others. Moreover, altering a capacity that decreases reactive aggression, which may be desired, might also decrease one's willingness to punish those who act violently, which may not be desired. So, in order for individuals to make autonomous decisions regarding moral enhancement, it is critical to understand how altering a capacity will alter moral behavior in ways that are either desirable or undesirable.

#### Permissible vs. obligatory moral enhancement

Certain proponents have argued that moral enhancement should be required, or obligatory (Persson and Savulescu, 2013, 2014). They argue that technological advances have created tools that can cause human suffering on catastrophic levels, ranging from climate instability to weapons of mass destruction, similar to historical arguments in favor of moral enhancement (Delgado, 1969). Without increasing our moral capacities, it is argued that humanity faces inevitable destruction. In order to prevent such destruction, mandatory moral enhancement is required. Such requirements clearly violate one's autonomy, but the impending moral catastrophe outweighs this loss of personal liberties.

Again, such an argument assumes that one intervention can be used to enhance all moral decisions, without considering the mechanism by which such enhancement must occur. The evidence presented from non-invasive brain stimulation instead shows that only specific cognitive-affective capacities contributing to moral behavior are likely to be enhanced, affecting some but not all behaviors, and not always in the desired direction. For example, improving empathy may lead to improved moral behavior toward one's in-group, but more harsh retaliation against those outside of one's group. Conversely, increasing cognitive processes contributing to utilitarian reasoning to consider the interests of many groups of persons might come at the expense of decreasing one's aversion to harming persons in general. Given this reality, it is unlikely that moral biomedical enhancement will alter behavior to the extent necessary to requiring individuals to use moral enhancing technologies. Proponents of moral enhancement agree that such technologies are unlikely in the near future but should be possible eventually (Persson and Savulescu, 2013, 2014). However, it is difficult to imagine how such a technology could ever be developed given our current understanding of moral psychology.

#### Freedom of choice and authenticity

Harris (2011, 2013) has argued that moral enhancement would reduce an individual's freedom of choice, even if that choice is to act immorally. Invoking Milton's *Paradise Lost*, he argues that part of what makes humanity worthwhile is the freedom to fall from grace (Harris, 2011). Again, this implies that moral enhancement would make it impossible to act immorally, while the evidence from non-invasive brain stimulation suggests that such universal improvement is unlikely. More likely, moral enhancement will alter an individual's tendencies to make certain moral decisions but not others. Because the decision to enhance would be free, doing so would improve an individual's ability to act in accordance with what they deem to be morally appropriate. While preferences might shift, the choice would remain free.

This argument is perhaps similar to the authenticity debate in cognitive enhancement, where some have expressed concern that enhancement would taint the authentic, or true, self (Parens, 2005). One could similarly argue that moral enhancement would lead to inauthentic person, even if morally superior. This debate centers on whether authenticity is considered an innate, static gift that should be appreciated and unchanged, or an ultimate goal that one must aspire toward Parens (2005). According to the latter definition, neither cognitive nor moral enhancement threatens authenticity.

#### Moral plurality

Part of the difficulty in understanding moral enhancement is that many moral challenges do not have clear solutions. So while there is agreement that improving memory, multi-tasking, and executive functions are indicators of cognitive enhancement, it is not clear how moral enhancement is supposed to affect our views on controversial topics like abortion, distributed justice, or choosing between the welfare of different groups of persons. A more reasonable goal would be to enhance certain moral motivations that are universally agreed upon, which research in non-invasive brain stimulation has shown to be theoretically possible. However, enhancing a single capacity is unlikely to lead to what one would consider a superior moral decision in every instance.

That being said, it is reasonable that enhancing certain cognitive-affective processes would lead to improved moral behavior on average (Douglas, 2016). Moreover, those with relatively low baseline capacities for certain moral motivations might show a significant benefit from moral enhancement. By understanding the mechanism and implications of moral enhancement using non-invasive brain stimulation, a rational but ethical implementation is possible.

## Conclusions

In conclusion, limited but growing evidence thus far suggests that brain stimulation can modulate specific cognitive-affective processes involved in moral behavior, making moral enhancement possible. However, rather than improving one single moral capacity, brain stimulation alters specific neuropsychological processes contributing to moral behavior. Enhancement of these processes can lead to morally enhanced behavior in some situations, but less morally desirable behavior in other circumstances. This influences the ethical debate regarding moral enhancement, showing that technologies will be unlikely to change moral behavior to the extent required to make moral enhancement obligatory, or to raise concern regarding our freedom to act immorally. However, the more modest goal of improving our tendencies to act in accordance with our moral motivations is likely possible, and may be desirable for large numbers of people. Given the very real threats of weapons of mass destruction and other technologies noted by past and current scholars, there is a clear need to research moral interventions in greater depth. Such research is needed to determine the implications of enhancing certain capacities contributing to moral behavior so that informed, rational debates regarding the use of moral enhancement are possible.

## Author contributions

RD for study concept and design and for writing the manuscript, AP for critical revisions of the manuscript.

## Funding

This work was supported by funding from the Sidney R. Baer, Jr. Foundation (RD, AP), the NIH (R01HD069776, R01NS073601, R21 NS082870, R21 MH099196, R21 NS085491, R21 HD07616 to AP), the Football Players Health Study at Harvard University (AP), and Harvard Catalyst. The Harvard Clinical and Translational Science Center (NCRR and the NCATS NIH, UL1 RR025758 to AP).

### Conflict of interest statement

The authors declare that the research was conducted in the absence of any commercial or financial relationships that could be construed as a potential conflict of interest.
